# The Role of Long Non-Coding RNAs in Intracranial Aneurysms and Subarachnoid Hemorrhage

**DOI:** 10.3390/life10090155

**Published:** 2020-08-20

**Authors:** Ilgiz Gareev, Ozal Beylerli, Gjumrakch Aliev, Valentin Pavlov, Adel Izmailov, Yiwei Zhang, Yanchao Liang, Guang Yang

**Affiliations:** 1 Bashkir State Medical University, 450008 Ufa, Republic of Bashkortostan, Russia; ilgiz_gareev@mail.ru (I.G.); obeylerli@mail.ru (O.B.); ilgizclassic@gmail.com (V.P.); 2 Sechenov First Moscow State Medical University (Sechenov University), 119146 Moscow, Russia; morfolhum@mail.ru; 3 Research Institute of Human Morphology, Russian Academy of Medical Science, 117418 Moscow, Russia; 4 Institute of Physiologically Active Compounds, Russian Academy of Sciences, Chernogolovka, 142432 Moscow, Russia; 5 GALLY International Research Institute, San Antonio, TX 78229, USA; 6 Regional Clinical Oncology Center, 450054 Ufa, Republic of Bashkortostan, Russia; izmailov75@mail.ru; 7 Harbin Medical University, Harbin 150081, China; dr_daming_zhang@163.com or; 8 Department of Neurosurgery, the First Affiliated Hospital of Harbin Medical University, Harbin 150001, China; liangyanchao@hrbmu.edu.cn; 9 Institute of Brain Science, Harbin Medical University, Harbin 150001, China

**Keywords:** intracranial aneurysm, long non-coding RNAs, subarachnoid hemorrhage, pathogenesis, biomarker, therapy

## Abstract

Intracranial aneurysms (IAs) represent the most complex and relevant problem of modern neurology and neurosurgery. They serve as one of the main causes of non-traumatic subarachnoid hemorrhage (SAH), causing up to 85% of all cases of intracranial hemorrhage, which is associated with frequent disability and high mortality among patients. Unfortunately, the molecular mechanisms of the development and rupture of IAs are still under study. Long non-coding RNAs (lncRNAs) are non-coding RNAs that typically have a length of more than 200 nucleotides. It is known that lncRNAs regulate many processes, such as transcription, translation, cell differentiation, regulation of gene expression, and regulation of the cell cycle. In recent years, a lot of evidence has established their role in human diseases from oncology to cardiovascular disease. Recent studies have shown that lncRNAs may be involved in the pathogenesis of IAs. The study of lncRNAs and its targets in various pathological conditions of a person is a rapidly developing field, and it is likely that the knowledge obtained from these studies regarding the pathogenesis of intracranial aneurysms will have the potential to use lncRNAs in therapy, as well as in the diagnosis and prediction of high aneurysms risk of rupture.

## 1. Introduction

Intracranial aneurysms (IAs) occur in 2% to 3% of the general population and are characterized by localized structural deterioration of the arterial wall, with loss of the internal elastic lamina and disruption of the media [1]. Rupture of IAs can cause 85% of subarachnoid hemorrhage (SAH), a death of 30–50% of the cases, and a severe disability in 30% of the cases [1,2]. At present, the treatment and prevention of intracranial aneurysms are still challenged due to their unknown pathogenesis. 

In response to the destruction of the internal elastic lamina and subsequent mechanical overload and tensile shear force, vascular smooth muscle cells (VSMCs) and fibroblasts synthesize collagen types I and V, which are the main molecular constituents of IAs. VSMCs, which perform contractile functions in the vessel wall, may initially migrate into the intima in response to endothelial damage. A change in the physiological location and phenotype of VSMCs towards the synthetic type can contribute to the restoration of the vessel wall through collagen synthesis, which leads to neointimal hyperplasia. In addition, prolonged hemodynamic shear stress on the vascular wall leads to degradation of the extracellular matrix (ECM), endothelial cells (ECs) dysfunction, and apoptosis or phenotypic modulation of VSMCs towards undifferentiated pro-inflammatory VSMCs. Once molecular mechanisms cannot compensate for the mechanical overload of the vessel wall and neointimal damage, cellular and humoral inflammatory responses become the main factors in the formation of IAs. These responses, which are mediated by inflammatory cytokines such as tumor necrosis factor (TNF), IL-1β and matrix metalloproteinases (MMPs), promote the influx of macrophages and permanent degradation of collagen and elastin fibers.

Long non-coding RNAs (lncRNAs) are frequently defined as non-protein-coding transcripts larger than 200 nucleotides. LncRNAs, typically transcribed by RNA polymerase II, are a very heterogeneous size group, some of which can span several tens of kilobases (kb). The genes of lncRNA share several characteristics with the protein-coding genes, such as similar epigenetic profiles, the presence of splicing and polyadenylation signals, as well as the size of exons and introns [3]. However, and compared to mRNAs, lncRNAs are generally more enriched in the nucleus and show lower retention of sequence, although some of them are strongly preserved. In addition, the lncRNAs are expressed more weakly than the protein-coding genes and their expression is remarkably specific to certain tissues. Depending on their position relative to the coding genes, lncRNAs can be divided into two broad categories: intergenic lncRNAs and intragenic lncRNAs. Intergenic lncRNAs, located by definition in unannotated regions of the genome, are generally called lincRNAs. They represent the best-studied class of lncRNA today. Intragenic lncRNAs, on the other hand, can be subdivided according to how they overlap the coding genes or their orientation with respect to them (antisense, intron, etc.). It should be noted that many intergenic lncRNA genes have a transcription initiation site close to that of a coding gene, with transcription occurring on the opposite strand (divergent transcription). Recently, a study has demonstrated that the genes associated with these divergent transcripts frequently encode transcriptional regulators involved in cell development and differentiation [3]. Finally, some lncRNAs overlap small RNAs, such as small nucleolar RNAs (snoRNAs) or miRNAs, with potential functional links, as in the case of regions subjected to genomic fingerprinting. Multiple lncRNAs contain repeating elements, such as long interspersed nuclear elements (LINE) or short interspersed nuclear elements (SINE), with potential functional implications. However, taken as a whole, lncRNAs do not have a conserved sequence or structure that might be indicative of a particular function. Therefore, most studies aimed at identifying potentially relevant lncRNAs in a given physiological or pathological context are based on co-expression or co-regulation analyses. LncRNAs are key players in the epigenetic, post-transcriptional, and translational coordination of gene expression in developmental and disease processes [3]. Recent studies have shown that lncRNAs are partially specific for tissue and cell type, suggesting their functional significance. The combination of ECM disruption, dysfunction of ECs, VSMCs phenotypic switching, overproduction of reactive oxygen species (ROS), inflammation, and immune responses, represents a dynamic process that leads to deterioration of the vascular wall and formation of IAs [1]. However, only a few reports cited here were able to provide evidence for a suspected molecular mechanism of action being mediated through lncRNAs. The detailed underlying mechanisms that regulate these key processes still need many more studies and involves lncRNAs. Obtaining a better understanding of the cellular mechanisms and regulatory networks driving IA development and progression is essential to identify novel therapeutic targets. In addition, lncRNAs can be detected in biological fluids, such as a plasma or serum samples, and they may potentially act as non-invasive biomarkers for the diagnosis and prognosis of IAs and SAH [4]. In this review, we will review relevant studies regarding lncRNAs, IAs, and SAH, and explain the complex relationship between them. We further discuss the clinical potential of lncRNAs for the development of novel diagnostic and therapeutic strategies.

## 2. LncRNAs and IAs

An increasing number of studies have demonstrated the direct role of lncRNAs in the pathogenesis of various human diseases including tumors, inflammatory, cardiovascular, and immunological diseases [3,5]. In many studies, the regulatory mechanism of lncRNAs in aortic aneurysms was also shown, which was directly associated with the formation, growth and dissection of the aorta (Table 1) [6,7,8,9,10,11,12,13,14,15,16,17,18,19]. In recent years, increasing studies have focused on role lncRNAs in the pathogenesis of IAs. For instance, lncRNAs have been reported to be upregulated in atherosclerotic and hypertension disease, which are important risk factors for the development of IAs (Table 2) [20,21,22,23,24,25,26]; by signaling to unique miRNAs or proteins, it may participate in multiple processes like VSMC phenotypic switching, inflammation activation, ECM disruption, endothelial dysfunction, cells necrosis, and overproduction of ROS, thus development, formation, growth, and rupture of IAs. LncRNAs molecular functions in IAs are very diverse. For instance, in aneurismal tissues from IA patients (12 ruptured IAs and 15 unruptured IAs), a microarray study indicated that 4129 lncRNAs (876 upregulated; 3253 downregulated) and 2926 mRNAs were significantly dysregulated. The authors further performed lncRNA-mRNA co-expression network analysis (Gene ontology (GO) and Kyoto Encyclopedia of Genes and Genomes (KEGG)) and the co-expression networks were represented in immune response, inflammatory response, the muscle contraction pathway, and the vascular smooth muscle contraction pathway. There were 24 lncRNAs that interacted with eight mRNAs in the vascular smooth muscle contraction, 10 lncRNAs interacted with 10 mRNAs in the GO term of immune response, seven lncRNAs interacted with seven mRNAs in the GO term of inflammatory response, and 31 lncRNAs interacted with nine mRNAs in the muscle contraction pathway [27].

ANRIL, also known as CDKN2B-AS, is a long non-coding RNA mostly localized in the nucleus, spanning 126.3 kb in the genome, and its spliced product is a 3834 bp RNA [28]. ANRIL is highly expressed in ECs and VSMCs and associated with a variety of human diseases, such as myocardial infarction, ischemic stroke. and aortic aneurysm, with single nucleotide polymorphisms (SNP) in this region [28]. There have been a lot of scholar studies that supported ANRIL multiple SNPs loci associated with IAs. For example, Chen et al. confirmed rs1333040 and rs6475606 associated with sporadic IA in the Chinese population [29]. Helgadottir et al. confirmed rs10757278 in ANRIL associated with IA in European individuals [30]. Low et al. found that rs10757272 in ANRIL was significantly associated with IAs in the Japanese population, and confirmed the role of rs10757278 [31]. Alg et al. also confirmed ANRIL SNP loci could lead to susceptibility of IAs on the basis of predecessors’ research results, and believe rs10757278 have significant effect on IA [32]. Additionally, studies indicate that the association between rs10757278 and IA was independent of hypertension and smoking. 

H19 is another lncRNA that is strongly associated with cardiovascular disease, such as high blood pressure, ischemic stroke and coronary artery disease [28]. H19 is a well-studied lncRNA that is important in cell differentiation and growth [33]. Chen et al. provided the first evidence those GWAS-discovered IA susceptibility loci BET1L rs2280543 may contribute to the risk of IA by interacting with H19 in the Chinese population [34]. 

Recent studies have shown that hypoxia-inducible factor 1α-antisense RNA 1 (HIF1A - AS1) may be involved in the formation of aortic aneurysms by regulating the proliferation and apoptosis of VSMCs [12,13,16]. Xu et al. reported that HIF1A - AS1 is involved in the pathogenesis of IAs [35]. In the present study, it was shown that increased expression of HIF1A - AS1 inhibits the proliferation of VSMCs and increases the expression of transforming growth factor β1 (TGF - β1). In addition, TGF-β signaling has been shown to be involved in the regulation of VSMCs proliferation, indicating potential involvement of TGF-β signaling in IA pathogenesis. TGF-β signaling under certain conditions fulfills its biological functions through interaction with specific lncRNAs, which play a critical role in various human diseases. 

Additionally, Man et al. showed that downregulation of lncRNA GASL1 might have a role in the pathogenesis of IA through its effects on VSMCs [36]. This study also showed that the actions of GASL1 in IA might be achieved through the interactions with TGF-β1 and its regulatory roles on the proliferation of VSMCs. 

Microarray analysis, using the aneurysm tissues and superficial temporal arteries (STAs) samples of patients with IA, indicated that 1518 lncRNAs were significantly dysregulated (413 were upregulated, and 1105 were downregulated) in aneurysm tissue [37]. In addition, the protein-coding mRNA profiles between the aneurysm tissues and STAs were also compared. In comparison with the mRNAs in the STAs samples, 2545 mRNAs were differentially expressed in aneurysm tissues (1150 were upregulated, and 1395 were downregulated). Furthermore, 4 lncRNAs and 4 mRNAs were confirmed by reverse transcription-quantitative polymerase chain reaction (qRT-PCR). Then, the authors employed lncRNA target-prediction program, KEGG and GO analysis to explore potential lncRNA functions. The results of further GO and KEGG indicated that lncRNAs were involved mainly in regulating immune/inflammatory processes/pathways (chemokine signaling, B-cell receptor signaling and cytokine–cytokine receptor interaction pathways) and vascular smooth muscle contraction (Myocardin, MYOCD; smooth muscle aortic alpha-actin, ACTA2; myosin heavy chain, MYH11; and myosin light chain 9, MYL9).

## 3. LncRNAs and SAH

Early brain injury (EBI) refers to the acute pathophysiological event that occurs within 2–3 days of SAH. EBI is one of the leading causes of disability and mortality worldwide in patients with SAH [38]. EBI is caused by blood brain barrier (BBB) disruption, brain edema, oxidative stress, inflammation, and neural cell death [39]. However, the molecular regulatory mechanism in EBI after SAH has been little studied. Evidence from some scientific studies shows that some lncRNAs are involved in the development of EBI following SAH through the regulation of broad signaling pathway, including inflammation.

Neuronal apoptosis usually occurs in EBI after SAH [40]. The p53 protein and neural growth factor (NGF) are important factors in many cellular processes, including cell apoptosis [41]. The p53 and NGF have been shown to be orchestrating proteins in the apoptotic pathways following a SAH [41,42]. LncRNAs have been shown to regulate p53 and NGF by affecting p53 and NGF mRNA stability and affecting transcription of their target genes. For example, Yang et al. reported that H19 regulates brain injury after SAH via miR-675 and let-7a by interacting with neuronal apoptosis induced by p53 and NGF [42]. The authors found that the administration of melatonin (MT) upregulated expression of H19, and H19 was shown to host miR-675, and miR-675 has been found to be a negative regulator of P53. In addition, H19 was found to be a competing non-coding RNA for let-7a, and let-7a was found to be a regulator of NGF by using a miRNA online database (www.mirdb.org). Liang et al. identified and functionally characterized lncRNA maternally expressed (MEG3) in neuronal cells of SAH rats whose expression is increased [43]. In addition, the expression of MEG3 was notably increased by lentiviral transfection in vitro. Overexpressed MEG3 significantly inhibited neuronal activity for 72 h. Meanwhile, overexpressed MEG3 resulted in increased neuronal apoptosis. Among this result, lncRNA promotes the neuronal apoptosis by inhibiting the Pi3k/Akt pathway [43]. Altered expression of MEG3 was observed to mediate ischemic neuronal death by activating p53 protein both in vitro and in vivo [44].

In the brain tissues of a rat SAH model, a microarray study indicated that 402 mRNAs and 208 lncRNAs were significantly dysregulated. Among these dysregulated mRNAs and lncRNAs, 221 upregulated and 181 downregulated mRNAs and 64 upregulated and 144 downregulated lncRNAs [45]. Furthermore, five lncRNAs (BC092207, MRuc008hvl, XR_006756, MRAK038897, and MRAK017168) were confirmed by qRT-PCR, where the expression levels of BC092207 and MRuc008hvl were significantly upregulated and the expression levels of XR_006756, MRAK038897, and MRAK017168 were markedly downregulated. Moreover, among these downregulated lncRNAs, MRAK038897 exhibited the most marked change. MRAK038897 is associated with ankyrin repeat and suppressor of cytokines signaling box 3 (ASB3), which is involved in the inflammatory process of EBI following SAH [45,46]. In addition, in order to better understand differentially expressed mRNA genes in this study, the KEGG and GO databases were used to analyze their potential biological functions. GO and KEGG pathway analysis demonstrated that these differentially expressed mRNA genes were involved in eight pathways, including ‘leishmaniasis’, ‘calcium signaling’, ‘tuberculosis’, ‘asthma’, ‘Staphylococcus aureus infection’, ‘chemical carcinogenesis’, ‘antigen processing and presentation’ and ‘neuroactive ligand-receptor interaction’. These pathways were mainly associated with inflammation, which has been confirmed to result in BBB disruption and brain edema [47]. In another study, Peng et al. identified 617 lncRNAs and 441 mRNAs that were aberrantly expressed in the mouse brain at 24 h after SAH [48]. KEGG and GO analysis revealed that these differentially expressed mRNA genes were involved in the inflammatory responses. Specifically, the authors found that among all lncRNAs, lncRNA fantom3_F730004F19 has the closest relationship with differentially expressed mRNA transcripts, which was correlated with CD14. The silencing of fantom3_F730004F19 resulted in reduced expression of CD14 and TLR4 at both the mRNA and protein levels. Moreover, knockdown of fantom3_F730004F19 attenuated inflammation in BV-2 microglia cells. These results suggest that fantom3_F730004F19 may have some specific effects on the pathological processes of EBI following SAH by regulating inflammation.

## 4. LncRNAs as Non-Invasive Biomarkers

Diagnosis of IAs mainly depends on imaging diagnostic methods, such as magnetic resonance angiogram (MRA), computed tomography angiography (CTA), and digital subtraction angiography (DSA) [49]. Despite the fact that preoperative diagnosis has improved in recent years, overall prognosis remains poor, and SAH remains a serious health problem. Therefore, efforts must be made to search for effective non-invasive biomarkers capable of identifying individuals at high risk of IAs development and its subsequent rupture. In this context, there has been a great deal of interest in circulating nucleic acids. Similar to circulating miRNAs, circulating lncRNAs were stably detectable in the human biofluids, such as whole blood, plasma/serum, or cerebrospinal fluid (CSF) [4,50]. In addition, there is need for a reliable, early, cost-effective, and non-invasive approach to screen IA patients in order to improve prognosis in SAH. Circulating lncRNAs could be packaged into microparticles, including microvesicles, exosomes, liposomes, and apoptotic bodies, to avoid being degraded [50]. Recently, several studies have indicated that circulating lncRNAs can be regarded as potential biomarkers for cardiovascular diseases (Table 3) [51,52,53,54,55,56,57,58], including IAs. For instance, for circulating long non-coding RNA-predicting development of IAs, metastasis-associated lung adenocarcinoma transcript 1 (MALAT-1) levels were independently associated with short disease-free survival (DFS) and poor overall survival (OS) for IA patients, and higher MALAT levels predicted a higher risk of death in IA patients [59]. In addition, the authors found that circulating MALAT1 expression was closely associated with hypertension history, aneurysm rupture and Hunt–Hess level. These results demonstrated that circulating MALAT1 expression in IA patients may be a novel biomarker for prognosis. qRT-PCR detection, using the serum of IA patients and healthy controls, revealed that circulating HIF1A-AS1 was greatly altered in IA patients [35]. The expression levels of HIF1A-AS1 were identified to be significantly increased in patients with IAs compared with healthy controls. The HIF1A-AS1 could differentiate IA patients from healthy controls with an area under the curve (AUC) of 0.879 (95% confidence interval (CI): 0.81–0.94). Therefore, circulating HIF1A-AS1 may serve as a potential diagnostic biomarker for IAs. Liang et al. tried to check the change in the expression of the circulating MEG3 in the CSF of SAH patients at four time points (1, 3, 5, and 7 days) [43]. They showed that the expression level of circulating MEG3 achieved a peak on the 3rd day after the clipping IA, and then gradually decreased to normal. Interesting, the expression level of circulating MEG3 was increased with the elevation of Hunt–Hess grade, which achieved the peak in SAH patients with Hunt–Hess IV-V, indicating that circulating MEG3 can be served as an indicator of SAH severity. Additionally, in another experiment of IAs, microarray assays showed that 797 circulating lncRNAs in the plasma were differentially expressed (519 upregulated and 278 downregulated) [60]. Of these, 10 circulating lncRNAs with the largest differential expression were selected for qRT-PCR validation. Plasma levels of lncRNA TCONS_00000200 and lncRNA ENST00000511927 were significantly higher in IA patients than in healthy controls with AUC 0.963 (95% CI: 0.919–1.000) and 0.804 (95% CI: 0.696–0.920), respectively. In addition, the authors argue that confirmed these results through qRT-PCR of venous blood samples from 10 patients with familial history of IA. Man et al. analyzed the relationship between serum GASL1 and TGF-β1 levels in patients with IA and healthy controls [36]. It has been shown that serum levels of GASL1 were significantly lower in patients with IAs than in healthy controls. Serum levels of TGF-β1 were significantly higher in patients with IAs than in healthy controls. ROC curve analysis showed that for serum GASL1, the AUC was 0.8820 (95% CI: 0.822–0.941), for serum TGF-β1, the AUC was 0.9420 (95% CI: 0.879–0.968). In this study, a significantly negative correlation was found between serum GASL1 and TGF-β1 levels in patients with IA.

## 5. LncRNA-Based Therapeutics

The characteristic pathology of IAs is characterized by progressive cerebral vessel wall dilation, promoted by dying VSMCs, “aberrant” proliferation, and migration of VSMCs, as well as impaired synthesis and degradation of ECM components, which at least partially is the result of transmural inflammation and its disruptive effect on cerebral vessel wall homeostasis [1]. Currently no conservative treatment approach exists that could slow down IA progression and protect the risk of rupture. The development and progression of various diseases, including IAs, can be associated both with activation of the expression of lncRNAs and with a decrease in their content in the cells [3,5]. Therefore, modern approaches to gene therapy are actively developing, aimed at activating and suppressing lncRNAs expression, as well as inhibiting their activity [61]. The methods of activating lncRNA expression include lncRNAs delivery by viral vectors, such as lentivirus, or by non-viral vectors such as inorganic or organic nanoparticles [62]. To suppress the expression of lncRNAs, approaches such as RNA interference using aptamers or small interfering RNA/short hairpin RNA (siRNA/shRNA), antisense oligonucleotides (ASOs), transcriptional repression, and gene editing tools such as CRISPR can be used [61,63]. It is known that overexpression of angiogenic factors, such as vascular endothelial growth factor A (VEGF-A), may be related to IA formation and rupture [64]. In addition, it has previously been discussed that cluster miR-143/miR-145 takes part in various biological processes associated with IA formation and the expression cluster miR-143/miR-145 is downregulated in IA tissues [65]. Gao et al. in their study showed that lncRNA metastasis-associated lung adenocarcinoma transcript 1 (MALAT1) and VEGF-A are upregulated and miR-143 is downregulated in IA tissues (rat models of IA and IA patients) [66]. Moreover, the authors demonstrated that a decrease in MALAT1 expression induced by short hairpin RNA (sh)-MALAT1 interference inhibited apoptosis and promoted the viability of ECs in IA by modulating miR-143/VEGF-A axis. In addition, after injection of (sh)-MALAT1 into the right cerebral ventricle of rats, a decrease in blood pressure and suppression of serum endothelin 1 (ET-1), von Willebrand factor (VWF), and matrix metalloproteinase-9 (MMP-9) expression occurred. The findings in this study partially disclose the pathogenesis of IA initiation and progression. Therefore, MALAT1 represents a potential target for novel IA treatment strategies. In another study, Ren et al. demonstrated that the upregulation of MALAT1 might function to downregulate miR-145, which further induces VEGF-A levels in oxygen–glucose deprivation (OGD)-induced brain microvascular endothelial cells (BMECs) [67].

Despite intensive studies of lncRNAs in cardiovascular disease, there is no lncRNA-based therapeutics entering clinical trials at present in this area. There are several obstacles facing the development of lncRNA-based therapeutics for IAs. One of the obstacles impeding development of lncRNA-based therapeutics is low delivery efficacy to the brain vasculature and the likely need for repeated delivery [68]. Second, the functions and mechanisms used by lncRNAs are much more complex and diversified than other non-coding RNAs [3]. In spite of recent studies, lncRNAs are still largely an “unknown” with regard to their cellular functions and molecular mechanisms of pathogenesis in IAs. Third, the majority of lncRNAs that localize to the nucleus are thought to act as epigenetic regulators [3,69]. This feature makes it hard to target lncRNAs using siRNA/shRNA, which is a potential therapeutic strategy. Fourthly, challenges for lncRNAs delivery remain with respect to their efficiency and tissue specificity [70]. While viral vectors induce immunogenicity, challenges for delivery lncRNAs may be overcome using chemical functionalization of nanoparticles surface targeted to specific ligands overexpressed by cells in the cerebral vessel wall in response to relevant stimuli [68,71]. In addition, most lncRNAs lack conservation between species, which restricts the utility of preclinical animal models. One possible strategy to overcome these issues is to identify the direct targets of aneurysm-associated lncRNAs and use preclinical animal models to assess the therapeutic role of these targets in IA pathogenesis and/or use innovative human-based model systems in vitro or ex vivo. With more and intensive studies of lncRNAs in vitro preclinical animal models and human patients, the application of lncRNA-based therapeutics in the clinic can become a reality.

## 6. Conclusions

**Figure 1:**
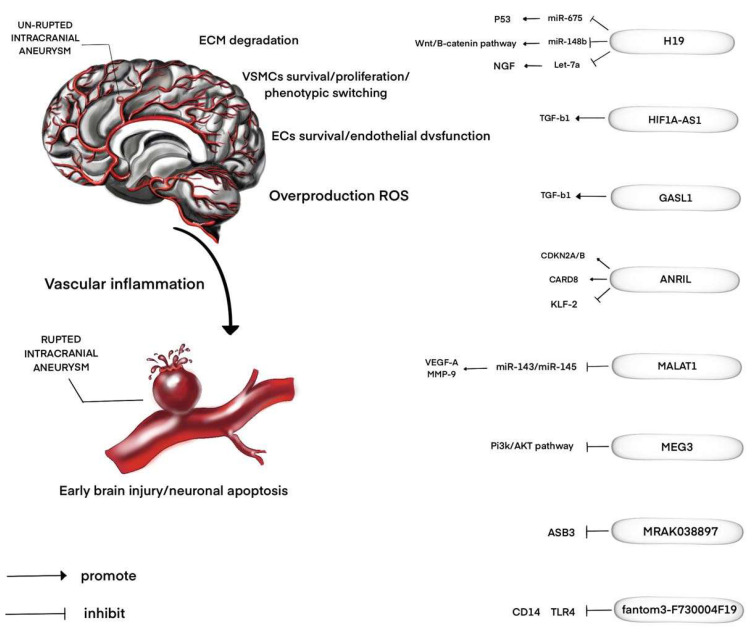
Long non-coding RNAs (lncRNAs) regulating intracranial aneurysm (IA) formation or progression. Selected targets that were shown to contribute to the effects of the lncRNAs in IA formation are shown. VSMCs, vascular smooth muscle cells; ECM, extracellular matrix, ECs, endothelial cells; ROS, reactive oxygen species; miR, microRNA; TGF-β1, transforming growth factor β1; MMP-9, matrix metalloproteinases 9; CD14, cluster of differentiation 14; TLR4, toll-like receptor 4; ASB3, ankyrin repeat and SOCS box containing 3; Pi3k, phosphoinositide 3-kinase; AKT, serine/threonine kinase; VEGF-A, vascular endothelial growth factor A; KLF-2, kruppel-like factor 2; CARD8, Caspase recruitment domain-containing protein 8; CDKN2A/B, cyclin-dependent kinase inhibitor 2A/B; NGF, neural growth factor; HIF1A - AS1, hypoxia-inducible factor 1α-antisense RNA 1; GASL1, growth-arrest-associated lncRNA 1; MEG3, Maternally Expressed 3; MALAT1, metastasis associated lung adenocarcinoma transcript 1

Our understanding of the molecular pathophysiology of IAs and SAH is improving very rapidly but is still limited. LncRNAs in IAs have emerged as a new research area (Figure 1). Only a few studies have attempted to correlate changes in the expression levels of lncRNAs and their gene targets with IAs and SAH in vitro and in vivo, in order to elucidate the mechanisms of pathogenesis. The results of these studies allow a better understanding of the processes associated with the formation, development, and rupture of IAs, and how risk factors, such as hypertension, predispose people to IAs. In addition, we suggested that lncRNAs would function as novel non-invasive biomarkers to predict the IAs and SAH, and may yield new therapies in the future.

## Figures and Tables

**Table 1:** LncRNAs involved in aortic aneurysms.

LncRNA	Type of Aneurysm	Gene-Target	Related Functions	Sample Studied	Cellular Origin	Regulation	References
LOXL1-AS	TAA	Giver	Promotes apoptosis and inhibit VSMCs proliferation	HAoSMCs, human aorta tissue	VSMCs	Up	[6]
HOTAIR	TAA	ALOX15B, AZU1, PIP, TBX1, VIL1, KRT20, PRAMEF22 MMP-8, KERA, CCBE1	Promotes apoptosis and inhibit VSMCs proliferation ECM remodeling (downregulates collagen type I and III expressions)	HAoSMCs, human aorta tissue, CABG tissue	VSMCs	Down	[7]
AK056155	AAA	TGF-β, PI3K/Akt	Development of LDS and aortic aneurysm	HUVECs, human serum	ECs	Up	[8]
HLTF-5	TAA	MMP9	ECM remodeling, VSMC phenotype switching	Human aorta tissue	VSMCs	Up	[9]
MIAT	TAA	MiR-145, PI3K/Akt/Bcl-2	Improves the viability and inhibits VSMCs apoptosis	Primary hVSMCs, human aorta tissue	VSMCs	Up	[10]
MALAT1	AAA	NADPH NOX2 and IL-6	Promotes AAA development	HAoSMCs, human aorta tissue	ECs	Up	[11]
HIF1A-AS1A	TAAA	Caspase-3, caspase-8, and Bcl2	Reduces apoptosis of VSMCs	HAoSMCs, human serum	VSMCs	Up	[12,13]
MALAT1	TAA	HDAC9 and BRG1	VSMCs dysfunction and ECM disruption (increase the activity of MMP2 and MMP9)	Primary hVSMCs, human aorta tissue	VSMCs	Up	[14]
PVT1	AAA	MMP2, MMP9, TNF-α, IL-1β, and IL-6	Suppresses VSMCs apoptosis, ECM disruption (decrease the activity of MMP2 and MMP9), and serum pro-inflammatory cytokines (TNF-α, IL-1β, and IL-6)	Primary mVSMCs, human and mouse aorta tissue	VSMCs	Down	[15]
HIF1A-AS1	TAA	BRG1	Promotes apoptosis and inhibit VSMCs proliferation	Primary hVSMCs, human aorta tissue	VSMCs	Up	[16]
GAS5	AAA	MiR-21 /PTEN/Akt, YBX1	Promotes apoptosis and inhibit VSMCs proliferation	HAoSMCs, human and mouse aorta tissue	VSMCs	Up	[17]
H19	AAA	HIF1α	Promotes apoptosis and inhibit VSMCs proliferation	HAoSMCs, human and mouse aorta tissue	VSMCs	Up	[18]
H19	AAA	Let-7a, MCR-1, IL-6,	Promotes aneurysm formation by enhancing vascular pro-inflammatory cytokines and enhancing macrophage infiltration.	Primary mVSMCs and mouse macrophages, human and mouse aorta tissue	VSMCs	Up	[19]

**Abbreviation:** AAA, abdominal aortic aneurysm; TAA, thoracic aortic aneurysm; TAAA, thoracoabdominal aortic aneurysm; LOXL1-AS1, LOXL1-antisense RNA; HOTAIR, HOX Transcript Antisense RNA; MIAT, myocardial infarction-associated transcript; MALAT1, metastasis-associated in lung adenocarcinoma transcript-1; PVT1, plasmacytoma variant translocation 1; HIF1A-AS1, antisense hypoxia inducible factor 1 alpha antisense RNA; GAS5, growth-arrest-specific transcript 5; miR, microRNA; ALOX15B, arachidonate 15-lipoxygenase type B; AZU1, azurocidin 1; PIP, prolactin induced protein; TBX1, T-box transcription factor 1; VIL1, villin 1; KRT20, keratin 20; PRAMEF22, PRAME family member 22; MMP, matrix metalloproteinase; KERA, keratocan; CCBE1, collagen and calcium-binding EGF domain-containing protein 1; TGF-β, transforming growth factor-β; PI3K, phosphoinositide 3-kinases; Akt, RAC-alpha serine/threonine-protein kinase; Bcl-2, B-cell lymphoma 2; PTEN, phosphatase and tensin homolog deleted on chromosome 10; YBX1, Y box binding protein-1; HIF1A, hypoxia-inducible factor 1-alpha; MCR-1, plasmid- mediated colistin resistance; IL-6, interleukin-6; HAoSMCs, human aortic smooth muscle cells; HUVECs, human umbilical vein endothelial cells; VSMCs, vascular smooth muscle cells; ECs, endothelial cells; ECM, extracellular matrix; mVSMC, mouse vascular smooth muscle cells; hVSMC, human vascular smooth muscle cells; LDS, Loeys–Dietz syndrome.

**Table 2:** List of lncRNAs potentially implicated in atherosclerosis and hypertension disease and their regulatory mechanisms.

LncRNA	Gene-Target	Disease	Regulation	Regulatory Effect of lncRNA	References
LEF1-AS1	miR-544a/PTEN axis	Atherosclerosis	Up	Regulates VSMCs proliferation and migration	[20]
lncRNA 430945	ROR2/RhoA	Atherosclerosis	Up	Promotes the proliferation and migration of VSMCs	[21]
lncRNA-ATB	TGF-β1 and caspase-3	Atherosclerosis	Up	Promotes apoptosis and inhibits proliferation of ECs	[22]
lncRNA AF131217.1	miR-128-3p/KLF4 axis	Atherosclerosis	Down	Reduces endothelial inflammation	[23]
lncRNA AK094457	PPARγ	Hypertension	Up	Increase Ang II-induced hypertension and endothelial dysfunction	[24]
MALAT1	Notch-1	Hypertension	Down	Alleviates the vascular lesion and remodeling: reduction relative expression of inflammation-related, endothelial function-related and oxidative stress-related factors; inhibit ECs apoptosis	[25]
MRAK048635_P1	Cyclin D1/E, CDK2/4, p-Rb, caspase3, PARP, α-SMA and calponin	Hypertension	Down	Induces VSMCs phenotypic switching from a contractile to a secretory phenotype. Promotes proliferation, migration and inhibits apoptosis of VSMCs	[26]

**Abbreviations:** TGF-β1, Transforming growth factor-β1; PPARγ, Peroxisome proliferator-activated receptor gamma; PTEN, Phosphatase and tensin homolog deleted on chromosome 10; ROR2, Receptor tyrosine kinase like orphan receptor 2; RhoA, Ras homolog family member A; TGF-β1, Transforming growth factor beta 1; KLF4, Krueppel-like factor 4; Cyclin D1/E, Cyclin D1/E; CDK2/4, Cyclin-dependent kinase 2/4; p-Rb, Retinoblastoma protein; PARP, Poly (ADP-ribose) polymerase; α-SMA, Alpha-smooth muscle actin; Notch-1, Notch homolog 1, translocation-associated; VSMCs, vascular smooth muscle cells; ECs, Endothelial cells; miR, microRNA.

**Table 3:** Circulating lncRNAs as possible biomarkers for cardiovascular diseases.

Disease	LncRNA	Sample	Regulation	Diagnostic	Prognostic	Sensitivity %	Specificity %	AUC	References
AIS	NEAT1	Plasma	Up	Yes	Yes	64.3	82.9	0.80	[51]
AIS	ANRIL	Plasma	Down	Yes	No	72.2	71.2	0.75	[52]
AMI	H19, MALAT1 and MIAT	PBMC	Up	Yes	No	Comb. 0.68	Comb. 0.76	Comb. 0.76	[53]
STEMI	LIPCAR	Plasma	Up	Yes	No	0.82	0.75	0.78	[54]
CAD	GAS5	Plasma	Down	Yes	No	0.78	0.92	0.97	[55]
HF	LIPCAR	Plasma	Up	No	Yes	/	/	/	[56]
DCM	LncRNA ENST00000507296	Plasma	Up	Yes	Yes	/	/	0.78	[57]
Athero-sclerosis	Exosomal HIF1A-AS1	Plasma	Up	Yes	No	0.86	0.82	0.75	[58]

**Abbreviations:** LncRNA, Long non-coding RNA; AUC, Area under ROC curve; AMI, Acute myocardial infarction; AIS, Acute ischemic stroke; HF, Heart failure; CAD, Coronary artery disease; DCM, Dilated cardiomyopathy; MALAT1, metastasis-associated in lung adenocarcinoma transcript-1; MIAT, myocardial infarction-associated transcript; GAS5, growth-arrest-specific transcript 5; NEAT1, Nuclear enriched abundant transcript 1; ANRIL CDKN2B-AS; LIPCAR, Long intergenic non-coding RNA predicting cardiac remodeling; HIF1A-AS1, Hypoxia inducible factor 1α-antisense RNA 1 Note: AUC ≥ 0.75 is considered diagnostically significant for the biomarker; Kaplan-Meier curves and log rank tests were used in articles to evaluate the prognostic significance of circulating miRNAs in cardiovascular disease; /, not mentioned in article.
